# Integrated metagenomics and metabolomics analysis reveals dynamic changes of microbiota and metabolic profile during fermentation of cigar tobacco (*Nicotiana tabacum* L.) leaves

**DOI:** 10.3389/fgene.2025.1662815

**Published:** 2025-11-10

**Authors:** Ting Yang, Xiaolu Lin, Rui Chen, Ruiqi Wang, Tong Li, Fulong Shen, Xiaowei Zhang, Lianghua Lai, Bing Lu, Jiliang Wei, Xiaofang Xie

**Affiliations:** 1 College of Life Sciences, Fujian Agriculture and Forestry University, Fuzhou, China; 2 Fujian Key Laboratory of Crop Breeding by Design, Fujian Agriculture and Forestry University, Fuzhou, China; 3 Institute of Tobacco Science, Longyan Tobacco Company, Longyan, China

**Keywords:** cigar tobacco leaves (CTLs), multi-omics, fermentation, microbial community, metabolites

## Abstract

Optimizing fermentation duration is critical for producing high-quality cigar tobacco leaves This study examines changes in microorganisms and metabolites during CTL fermentation at four time points: 0 days (T0), 25 days (T1), 50 days (T2), and 75 days (T3). We observed a decreasing trend in total soluble sugars, starch, total nitrogen, and nicotine levels as fermentation progressed. Notably, chemical components stabilized after T2 stages. The microbial community showed dynamic fluctuations, with alpha diversity indices (Shannon, ACE, Pielou’s evenness, and Chao-2) reaching equilibrium at T2 and maintaining stability thereafter. Dominant genera such as *Staphylococcus*, *Aspergillus*, *Sphingomonas*, and *Penicillium* persisted throughout the fermentation process. A total of 1801 metabolites were identified, with 584 showing differential expression across the fermentation periods. Notably, comparisons between T0 and T1, T2, and T3 revealed 218, 377, and 419 differentially expressed metabolites, respectively. KEGG enrichment analysis identified 28 co-existing metabolic pathways, seven of which are linked to cigar quality formation. Furthermore, 29 out of 47 differential metabolites significantly correlated with the eight dominant microbial genera. These findings indicate that the T2 stage achieves optimal balance between microbial activity and metabolite stabilization, providing a scientific basis for industrial process optimization.

## 1 Introduction

Cigars are a special product made from fermented CTLs. In comparison to traditional cigarettes, cigars are manufactured with uniform colors, unique flavors, low tar content, and pleasant aromas. These distinctive features have made them highly favored by consumers (Zheng et al., 2022a). However, as sales in the Chinese and international markets continue to increase, the shortage of high-quality cigar raw materials has become more prominent.

By optimizing the fermentation duration, not only can we ensure high-quality raw materials for cigar products, but also achieve cost savings. Generally, CTLs are recommended to undergo fermentation for a period of 4–7 weeks (Jie et al., 2014). For instance, Li et al. (2020) conducted a study and determined that the ideal fermentation duration for Shiyan, Hubei Province cigar No. 1 was 6 weeks. However, due to variations in origin, year, variety, grade and maturity of tobacco, the optimal fermentation time may vary (Zhou et al., 2016).

When determining the appropriate duration for CTLs fermentation, it is crucial to analyze the dynamic changes of essential substances throughout the entire process. This is because the fermentation process involves complex transformations of chemical substances, microorganisms, and metabolic products (Wang et al., 2007). Research has shown that the surfaces of CTLs harbor a significant number of bacteria and molds. The main bacterial species identified include *Bacillus*, *Pseudomonas* and *Sphingomonas*, while molds such as *Aspergillus* and *Penicillium* are predominantly present (Zhong et al., 2010; Zhou et al., 2020; Wang et al., 2023). During fermentation, *Bacillus* plays a crucial role in decomposing large molecules such as lignin and carotene through the secretion of specific enzymes. This enzymatic activity leads to the production of small aromatic substances (Dai et al., 2020), Additionally, *Pseudomonas* effectively degrades nicotine, contributing to improving the appearance, aroma, and resolving other defects in CTLs. Furthermore, metabolites including flavonoids, organic acids, alkaloids and amino acids play a vital role in determining the quality of cigar (Zhang et al., 2011). Among them all amino acids are closely related to cigar quality. Throughout fermentation duration as well as industrial processing and smoking stages both enzyme-catalyzed and non-enzyme-catalyzed reactions occur between amino acids and reduced sugars resulting in the formation of pyrrole heterocyclic compounds like pyrazine and furan. These compounds significantly impact the flavor profile of cigar. Moreover phenylalanine is capable of directly decomposing into flavor compounds such as benzyl alcohol or phenylethano (Hecht et al., 1977).

The advancement of microbial sequencing technologies (Diehl et al., 2013; Arsenic et al., 2015) and broadly targeted metabolomics (Raamsdonk et al., 2001) offer effective solutions for uncovering and analyzing the intricate microbial communities and metabolite profiles in various environments, including those relevant to the fermentation process of CTLs. For instance, Wang et al. (2018) utilized metagenome sequencing technology to investigate bacterial diversity on the surface of aged flue-cured tobacco. They discovered that the dominant bacterial community exhibited diverse functional characteristics involved in flavor substance synthesis and harmful compound degradation such as nicotine and nitrite. Furthermore, Zhang et al. (2013) and Zhao et al. (2013) employed Gas Chromatography-Mass Spectrometry (GC-MS) technology to determinate the metabolic profile of fresh tobacco leaves from different regions, identifying 20 distinct metabolites contributing to the differentiation of tobacco leaves from Yunnan, Guizhou, and Henan regions. Xia et al. (2014) conducted a comprehensive study on the metabolic profiles of tobacco leaves from various geographical sources by screening for several important metabolites associated with growing regions and climatic factors. These findings indicate that both microbial sequencing technology and metabolomic techniques can effectively analyze microorganism composition as well as to assess variations in microorganisms abundance across different ecological environments. However, contemporary research on CTL fermentation mainly relies on single omics methods. These methods failed to systematically analyze the relationship between microbial dynamic changes and substance transformation. In contrast, the multi omics comprehensive analysis method has significant advantages and helps to gain a more comprehensive understanding of the CTL fermentation process.

This study utilized continuous flow analysis technology, metagenomic sequencing, and widely targeted metabolomics to comprehensively analyze the dynamic changes in relevant chemical substances, microbial communities, and metabolic products during the fermentation process of CTLs. Furthermore, by employing KEGG functional enrichment and correlation analysis, we investigated the evolving patterns of major microbial communities and metabolites throughout the fermentation process. The objective of this research is to establish a solid theoretical foundation for determining the optimal fermentation time for CTLs.

## 2 Materials and methods

### 2.1 Plant materials and sampling

The study utilized the cigar variety of FX-01, which was cultivated in Longyan District of Fujian Province. Middle leaves (MLs) at positions 8 to 10 (numbered sequentially from the plant base upward) were selected for analysis. CTLs with a moisture content ranging from 21% to 24% were stacked in a fermentation chamber maintained at 38 °C. Samples were collected at specific time points during the fermentation process, including day 0 (T0), day 25 (T1), day 50 (T2), and day 75 (T3). The T0 samples served as the control group for comparison. At each fermentation stage, 18 leaves were carefully collected from six bundles of cigar tobacco leaves stacked in the middle of the fermentation chamber—three leaves from each bundle, with each bundle containing 30 cigar tobacco leaves. These leaves were pooled into three biological replicates, with each replicate consisting of six leaves from six distinct bundles. The leaf surface was wiped with sterilized cotton swabs at least thirty times. Two tobacco leaves were wiped with each cotton swab, repeated three times (three cotton swabs for six leaves). The used cotton swabs were placed into tubes and stored in liquid nitrogen for metagenome sequencing purposes. Each biological sample’s leaves were divided along the main vein into two parts: one-half was immediately frozen at −20 °C for chemical composition analysis, while the other half was quickly stored in liquid nitrogen for future metabolome detection.

### 2.2 Chemical composition analysis

Total soluble sugar, starch, total nitrogen, and nicotin content were assessed using a continuous flow method following the Chinese tobacco industry standard (YC/T 161-2002, YC/T 160-2002, YC/T 216-2007, and YC/T 159-2002). The measurement methods for all chemical compositions were in accordance with the description provided by the Chinese tobacco industry standard method (Grant et al., 2002).

### 2.3 Metagenome sequencing and data analysis

Microbial genome extraction, PCR amplification, library construction, sequencing, and routine data analysis were conducted by Biomarker Technologies (http://www.biomarker.com.cn/). Metagenome sequencing was performed on the Illumina Hiseq2500 platform following standard protocols utilizing the shotgun sequencing method. This approach enables the identification of both known and unknown microbes in a sample with high discriminatory power. The raw tags were filtered using Fastp software (Chen et al., 2018) to obtain clean tags. MEGAHIT software (Jiang et al., 2020) was used for metagenomic assembly with a filter for contig sequences shorter than 300 bp. The assembly results were evaluated using QUAST software (Alexey et al., 2013). MetaGeneMark software (Wenhan et al., 2010) (http://exon.gatech.edu/meta_gmhmmp.cgi, Version 3.26) was employed to identify coding regions with a minimum open reading frame (ORF) length of 100 bp and log-likelihood score >50. MMseqs2 software (Steinegger and Söding, 2017) (https://github.com/soedinglab/mmseqs2, Version 12-113e3) was used to remove redundancy based on a similarity threshold of 95% and coverage threshold of 90%. A BLAST comparison was then conducted between the protein sequences of non-redundant genes and those deposited in the Amino acid sequence of nonredundant protein (NR) and KEGG database, using an e-value cutoff of 1e-5 to search for similar sequences. Negative controls (sterile swabs without sample contact) were processed in parallel, and any taxa detected in controls were excluded from experimental samples using Decontam R package (v1.18.0) with prevalence-based threshold (threshold = 0.1).

The QIIME software (https://qiime2.org) was used for conducting alpha diversity and beta diversity analyses. To evaluate sequencing depth, dilution curves were plotted using R software (version 2.15.3). Python was employed to generate stacked bar graphs of species distribution at different taxonomic levels. Analysis of similarities (Anosim) based on Jaccard and Bray-Curtis distances with the GUniFrac package in R was conducted to compare microbial communities. Linear Discriminant Analysis Effect Size (LEfSe) analysis was performed using the LEfSe v20171228 tool in a Python 2 environment to identify potential biomarkers across different fermentation durations. All analyses were carried out on the BMK Cloud platform (www.biocloud.net).

### 2.4 Widely targeted metabolomics analysis

The biological samples were free-dried using a vacuum lyophilizer (Scientz-100F, China) and ground into fine powder (30 Hz, 1.5 min) with a grinder (MM 400, Retsch, Germany). Then, 50 mg of the powder was accurately weighed (MS105DU, Mettler Toledo, Swizerland) and dissolve in 1200 μL of 70% methanol extraction solution. The mixture was vortexed vigorously for 30s and centrifuged at 12,000 rpm for 10 min at 4 °C. The supernatant was carefully transferred to a new microcentrifuge tubes and filtered through a 0.22 μm microporous polytetrafluoroethlene (PTFE) membrane. The filtrate was stored in injection vials for subsequent metabolite analysis.

Metabolites profiling was performed using ultra-high performance liquid chromatography-tandem mass spectrometry (UPLC-MS/MS) on a UPLC system (ExionLC™ AD, Sciex) coupled with a triple quadrupole mass spectrometer (6500 QTRAP, Applied Biosystems). The mass spectrometer was operated in both positive and negative electrospray ionization (ESI) modes under the following parameters: ion spray voltage, ±5500 V; source temperature 550 °C; curtain gas 35 psi; collision energy, 10-50 eV(optimized per metabolite). Chromatographic separation was achieved on an Agilent SB-C18 column (1.8 μm, 2.1 mm × 10 mm) with a gradient elution program consisting of 0.1% formic acid in water (mobile phase A) and 0.1% formic acid in acetonitrile (mobile phase B) as follows: 0-1 min, 5% B; 1-8 min, 5%-95% B; 8-9 min, 95% B; 9-10 min, 5% B for column reequilibration. The flow rate was 0.35 ml/min and the injection volume was 2 uL. All solvents used were of chromatographic grade (methanol, formic acid, and acetonitrile; Sigma-Aldrich, United States). Metabolite detection and quantification were performed by Wuhan Metware Biotechnology Co., Ltd. (www.metware.cn).

Raw data were processed using MetWare’s proprietary software based on the MWDB database. Peak alignment, extraction, and integration were performed followed by minimum value imputation and normalization. Metabolites were identified by matching with standard mass spectra and retention times. For statistical analysis, hierarchical cluster analysis (HCA), principal component analysis (PCA), and orthogonal partial least squares-discriminant analysis (OPLS-DA) were conducted using R Software (v4.2.1) and GraphPad Prism (v9.1.0). Differential metabolites were screened based on variable importance in projection (VIP) ≥1 from the OPLS-DA model and absolute log_2_ fold change (FC) ≥1. A Venn diagram was used to illustrate the overlapping relationships among different comparison groups. Identified metabolites were annotated using the KEGG Compound database (https://www.kegg.jp/kegg/compound/) and mapped to pathways in the KEGG Pathway database (https://www.kegg.jp/kegg/pathway.html). Pathways containing significantly regulated metabolites underwent metabolite set enrichment analysis (MSEA), with significance assessed using p-values from hypergeometric tests.

### 2.5 Integrative analysis of metagenomics and metabolomics

To investigate the relationship between microbiota and metabolites, we conducted an integrated analysis. We prioritized common pathways identified in both metagenomics and metabolomics based on the results of KEGG pathways analysis. Subsequently, we highlighted the metabolic pathways that are closely related to CTLs quality within this shared set of metabolic pathways. Additionally, we analyzed the correlation between the differential metabolites within the key metabolic pathways and the microbial abundance of the Top10 using R version’s ggcor 0.9.8.1 package.

## 3 Results

### 3.1 Appearance and physiological indexes of CTLs undergo dynamic changes during different stages of fermentation

**FIGURE 1:**
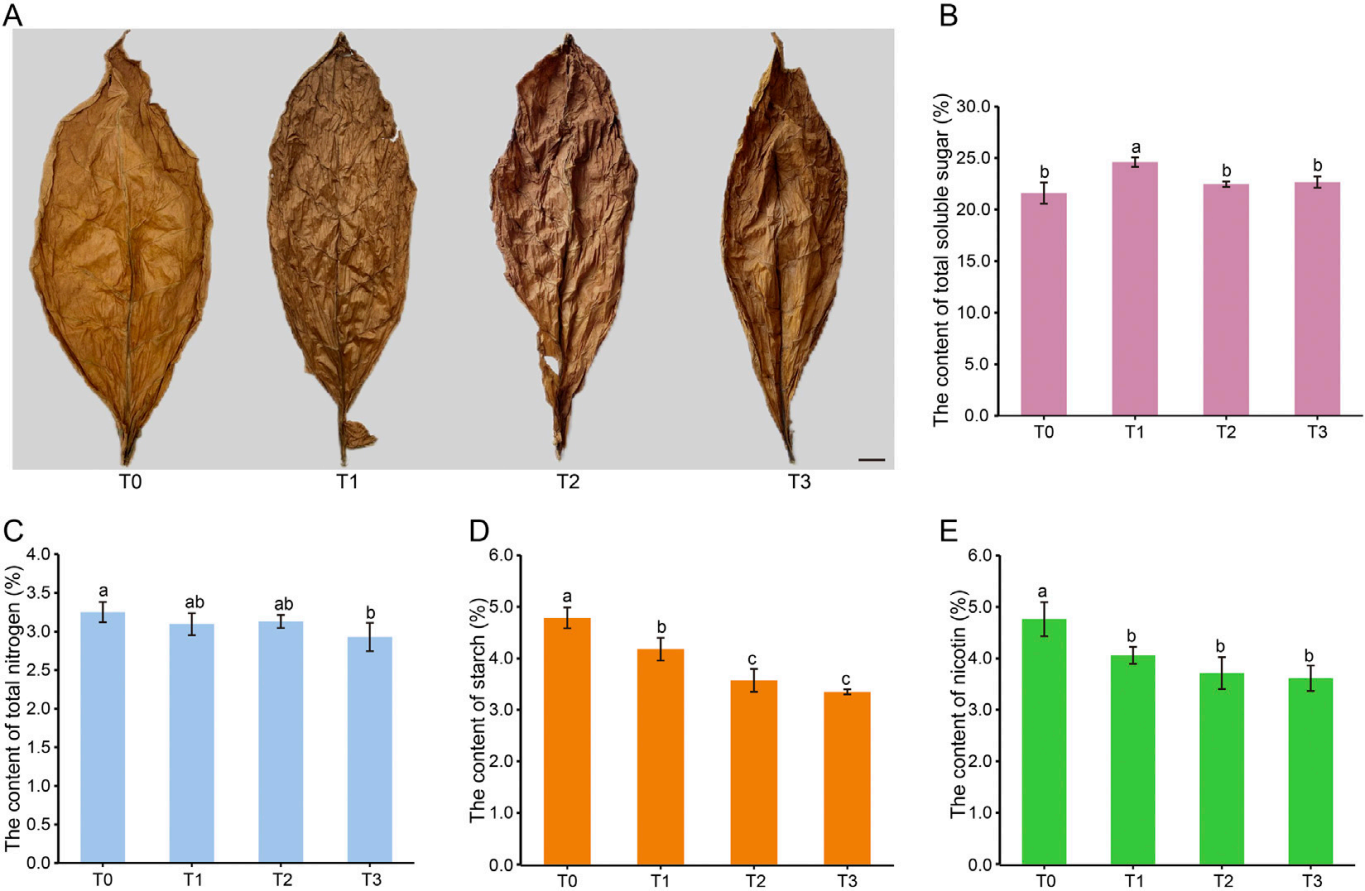
Appearance character **(A)** and chemical components **(B–E)** of CTLs at different fermentation periods. Note: T0 - T3 represents the fermentation of CTLs at the day 0, day 25, day 50 and day 75, respectively. Bar = 1 cm. Different lowercase letters after the same column of data indicate significant difference (*P* < 0.05).

During the fermentation process, cigar tobacco leaves undergo significant changes in appearance. As depicted in Figure 1A, there is a gradual decrease in the surface oil content of the leaves, resulting in creasing of the leaf surface. Moreover, the color of tobacco leaves progressively transforms from golden yellow at the beginning of fermentation (T0) to gray brown (T1), and eventually to a dark brown hue at T2 and T3. It is worth mentioning that slight cracking of the tobacco leaf surface begins from stage T1 onwards.

In order to study the dynamic changes in chemical composition related to cigar quality, this study measured the levels of starch, total soluble sugar, total nitrogen, and nicotine (Figures 1B–E). As fermentation progressed, the total soluble sugar content initially increased from 21.60% (T0) to 24.60% (T1), then gradually decreased to 22.66% (T3). Starch content showed a continuous decline from 4.79% (T0) to 3.35% (T3), while nicotine decreased from 4.76% (T0) to 3.62% (T3). In contrast, total nitrogen exhibited minimal variation, decreasing slightly from 3.25% (T0) to 2.93%. It is worth noting that there were no significant differences in the levels of these four chemical substances during the T2 and T3 stages. These results indicate that overall fermentation reduces macro-molecular chemical substances, with this reduction reaching a plateau during the T2 and T3 periods.

### 3.2 Overview of microbial community

**FIGURE 2:**
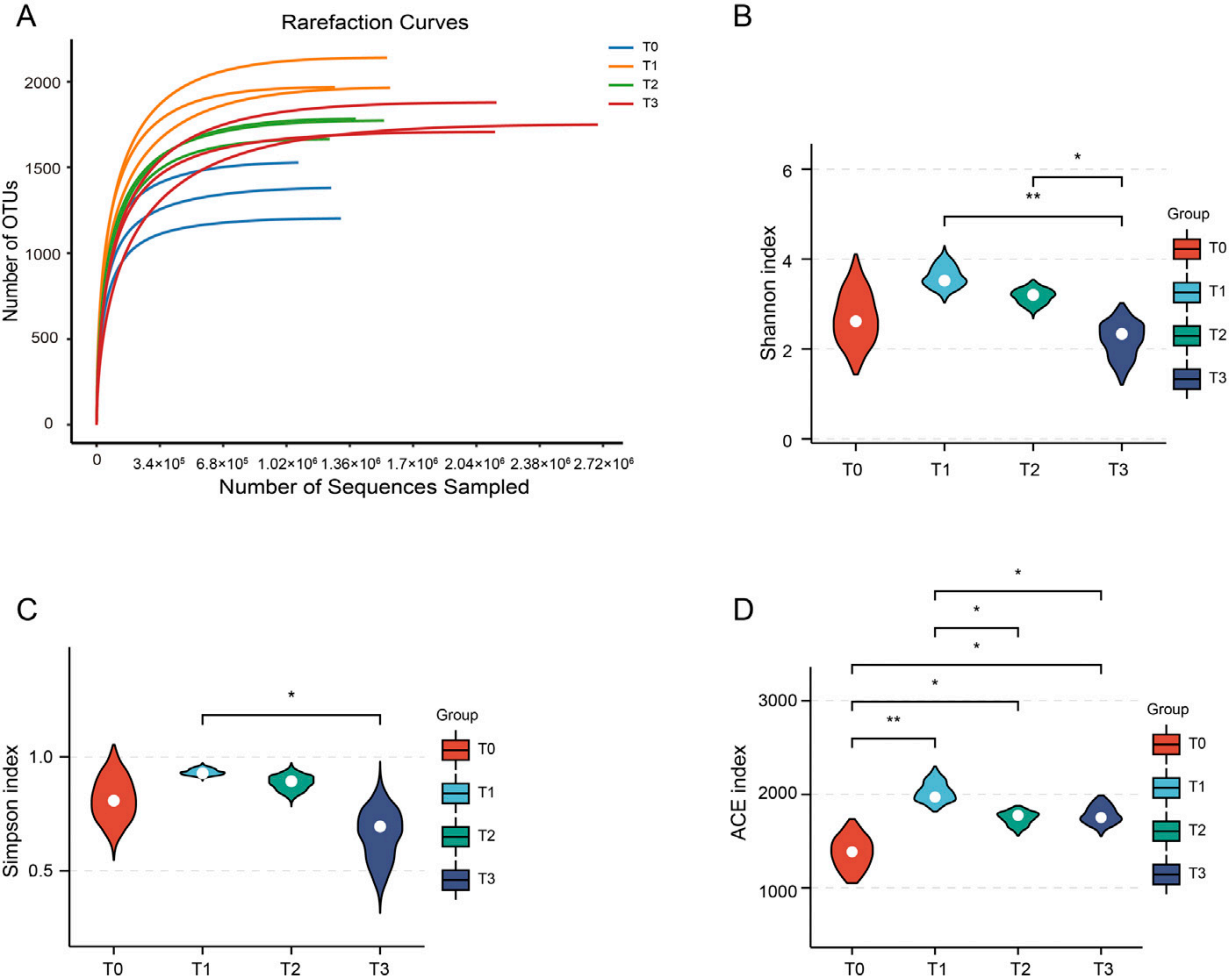
Dilution curves and alpha diversity of the CTLs. **(A)** Dilution curves for the four fermentation stages. **(B–D)** The alpha diversity of the CTLs microbiota is based on the Shannon, Simpson and ACE index, respectively. *P < 0.05, **P < 0.01.

To assess the dynamic change patterns of CTLs microbial communities at different fermentation stages, a total of 12 samples were analyzed using metagenomic sequencing technology (PRJNA1190085). After quality control, we obtained a total of 508,766,922 clean reads with each sample’s data exceeding 6.0 Gb. Following sequence assembly, we obtained 7,734,288 contigs with more than 40,000 contigs in each sample and Q30 greater than 93%. The assembly results demonstrated good assembly quality as indicated by N50 values exceeding 711 bp and a maximum value of 1321 bp (Supplementary Table S1). The gene prediction analysis revealed a range of 318,856 to 621,394 identified genes in each sample. Additionally, the average length of individual genes varied from 355 to 415 base pairs across the samples. The dilution curve showed a flat trend (Figure 2A), indicating comprehensive OTU coverage provided by deep sequencing.

Analysis of beta diversity in the samples revealed significant differences in community structure between different fermentation time points (Supplementary Figure S1). To assess the alpha diversity of the surface microbial community in CTLs, we examined the Shannon, Simpson, ACE, pielous_evenness and Chao-2 indices (Figures 2B–D; Supplementary Figure S2). The findings revealed that throughout the extended fermentation process, there was an initial increase in microbial diversity on CTLs’ surface followed by a gradual decrease. In T2 and T3 stages, apart from Shannon index, other indices demonstrated similar levels of microbial diversity as observed in the T1 stage where no significant difference was detected.

### 3.3 Taxonomic diversity and LEfSe difference of CTLs microbiota

**FIGURE 3:**
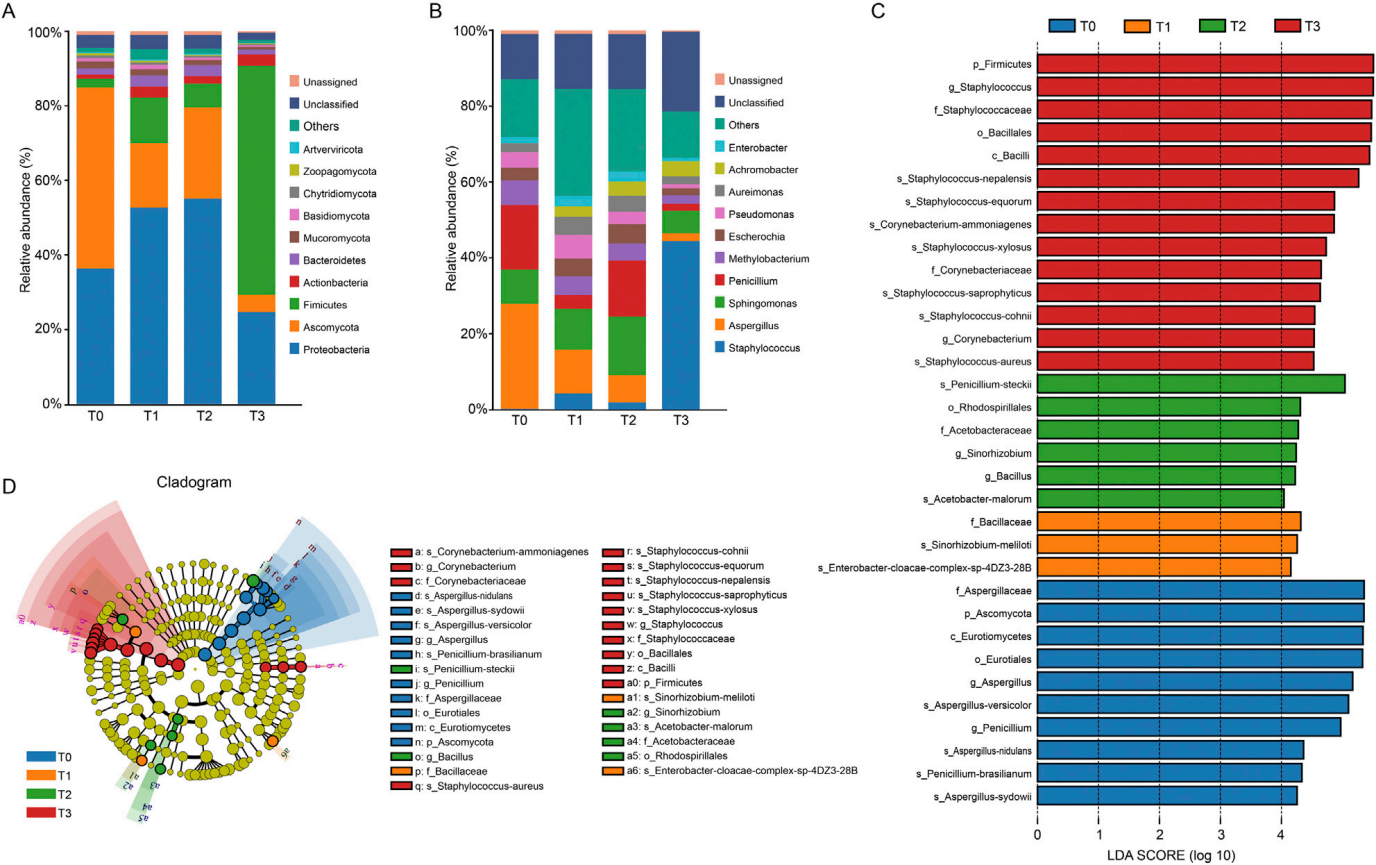
Taxonomic diversity and LEfSe different analysis of CTLs microbiota. Dominant microbial community composition at the phylum level **(A)** and the genus level **(B)** of the top 10. Unassigned refers to species that have not been annotated in the species annotation database; Unclassified refers to species that have been annotated by the species annotation database, but lack relevant information at this taxonomic level; others includes the sum of all the rest phyla (relative abundance, 0.01% each) generated by metagenomic data. **(C,D)** Evolutionary branching diagram based on LEfSe analysis (LDA score >4, *P* < 0.05). The circles from inner to outer represent taxonomic levels: phylum → class → order → family → genus. Colored nodes indicate taxa significantly enriched in T0 (blue), T1 (orange), T2 (green), or T3 (red).

During the fermentation process, the main microbiota at the phylum level were *Proteobacteria*, *Firmicutes*, and *Ascomycota*. Among them, the population of *Proteobacteria* initially increased from 38.16% (T0) to 57.78% (T2), then sharply declined to 25.31% (T3). Conversely, *Firmicutes* exhibited a sustained rise from 2.47% (T0) to 62.85% (T3) while *Ascomycota* dramatically decreased from 50.69% (T0) to 4.77% (T3). At the genus level, *Staphylococcus*, *Asperigullus*, *Sphingomonas* and *Penicillium* were the dominant microbiota. *Staphylococcus* exhibited explosive growth from **0.21% (T0)** to **56.55% (T3)**, while *Aspergillus* declined sharply from **31.46% (T0)** to **2.58% (T3)**. *Sphingomonas* showed a transient dominance at T2 (**18.18%**), and *Penicillium* fluctuated with peaks at T0 (**19.32%**) and T2 (**17.58%**) (Figures 3A,B).

To explore the microbial biomarkers of CTLs across fermentation duration, LEfSe analysis was performed with a linear discriminant analysis (LDA) threshold of 4 LDA and statistical significance (*P* < 0.05). The evolutionary branching diagram (Figures 3C,D) illustrates taxonomic relationships from phylum to genus levels (inner to outer circles), with colored nodes indicating taxa significantly enriched in specific stages (T0 - T3). A total of 33 microbial biomarkers were identified spanning 4 phyla, 5 classes, 6 orders, 7 families and 8 genera, with stage-specific counts as follows: T0 (10), T1 (3), T2 (6), and T3 (14). This U-shaped trend in biomarker abundance suggests initial community homogenization followed by divergence in later fermentation stages.

### 3.4 Metabolic profiling

**FIGURE 4:**
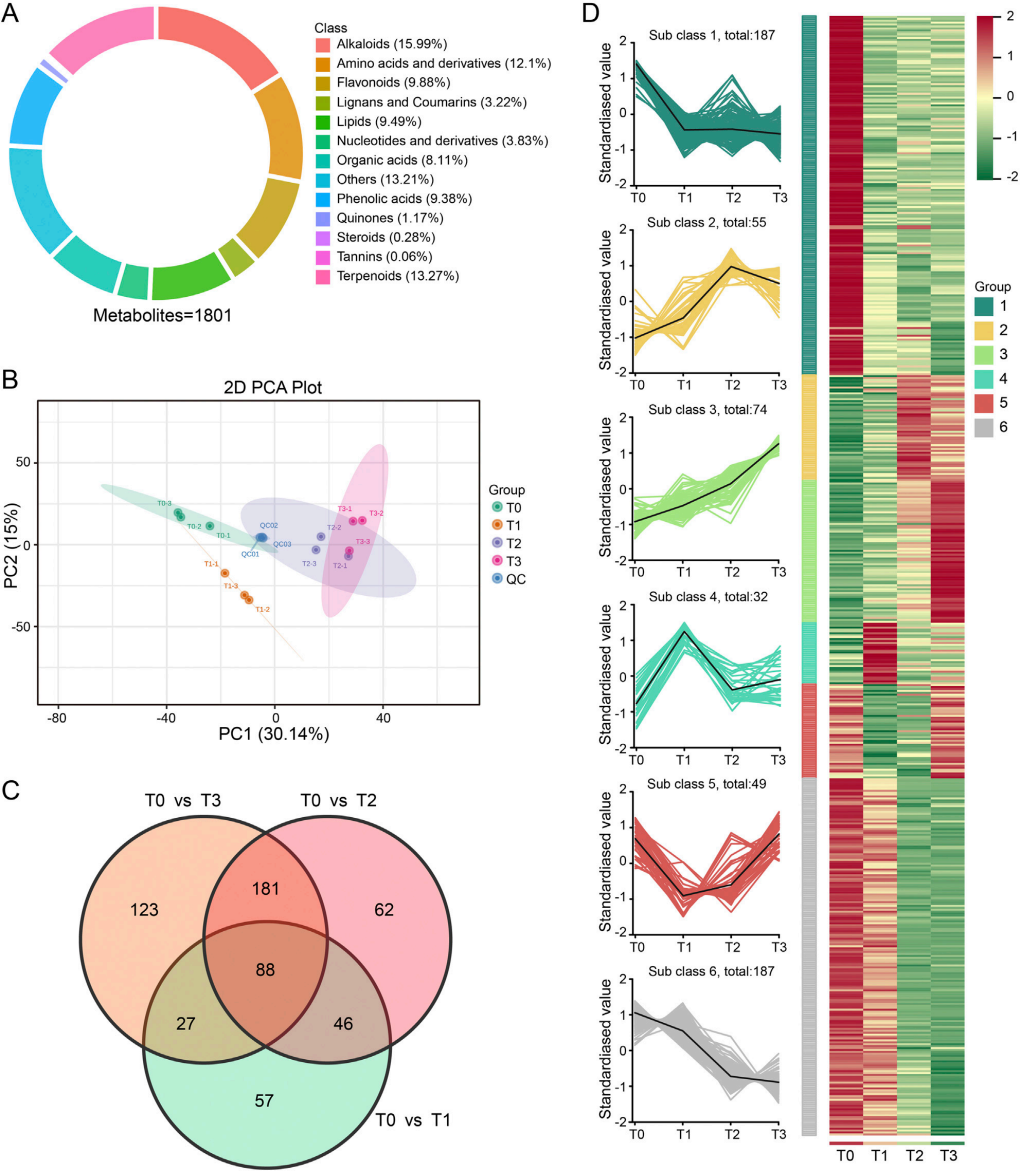
Analysis of CTLs metabolic profiles at 4 fermentation stages. **(A)** Classification of the 1801 metabolites of CTLs samples. **(B)** Score scatter plot for principal component analysis (PCA) model. **(C)** Venn diagram showing the numbers of common and specific differential metabolites among the different comparisons. **(D)** Line charts plot of K-means clustering of differential metabolites and visualization of clustering heatmap.

To investigate the impact of different fermentation times on metabolites, a comprehensive metabolic profiling was conducted on samples T0, T1, T2, and T3 using UPLC-MS/MS, and a total of 1801 metabolites were identified, including alkaloids (15.99%), terpenoids (13.27%), amino acid and its derivatives (12.1%), flavonoids (9.88%), lipids (9.49%), phenolic acids (9.38%), organic acid (8.11%), nucleotide and its derivatives (3.83%), lignans and coumarins (3.22%), quinones (1.17%), steroids (0.28%), tannins (0.06%), and others (13.21%) (Figure 4A).

Principal component analysis (PCA) was used to assess the reliability of the identification results and to examine the overall variations in compound composition among the 12 samples. The first and second principal components accounted for 30.14% and 15.00% of the total variation, respectively (Figure 4B). The PCA plot clearly demonstrated distinct clustering patterns among the samples corresponding to different fermentation stages. Additionally, the samples were primarily segregated based on three biological replicates, with the exception of T2 and T3 samples, which exhibited a slight tendency to aggregate. This result was consistent with the result of the hierarchical cluster analysis (HCA) (Supplementary Figure S3), indicating that there is substantial variation in metabolite levels across various fermentation periods.

The *Orthogonal Partial Least* Squares-Discriminant Analysis (OPLS-DA) model (Supplementary Figure S4) was used to identify differential metabolites among the three comparison groups. A total of 584 differential metabolites were detected, which were unevenly distributed across the three comparisons, namely, T0 vs. T1 (218), T0 vs. T2 (377) and T0 vs. T3 (419). Among them, 88 metabolites were found to be overlapping across multiple comparisons metabolites (Figure 4C; Supplementary Table S3). Further K-means cluster analysis showed that these 584 differential metabolites were grouped into 6 profiles (Figure 4D). The relative content of metabolites within each profile exhibited similarities in their clustering. Notable, profile 1 and 6 contained the largest sets of metabolites, each comprising 187 metabolites, followed by profile 3 (74 metabolites) and profile 2 (55 metabolites), respectively. In addition, the metabolites content included in profile 1 and 6 displayed a downward trend during CTLs fermentation. In contrast, 74 metabolites included in profile 3 showed continuously upregulated.

### 3.5 Combination analysis of metabolomic and metagenomic

**FIGURE 5:**
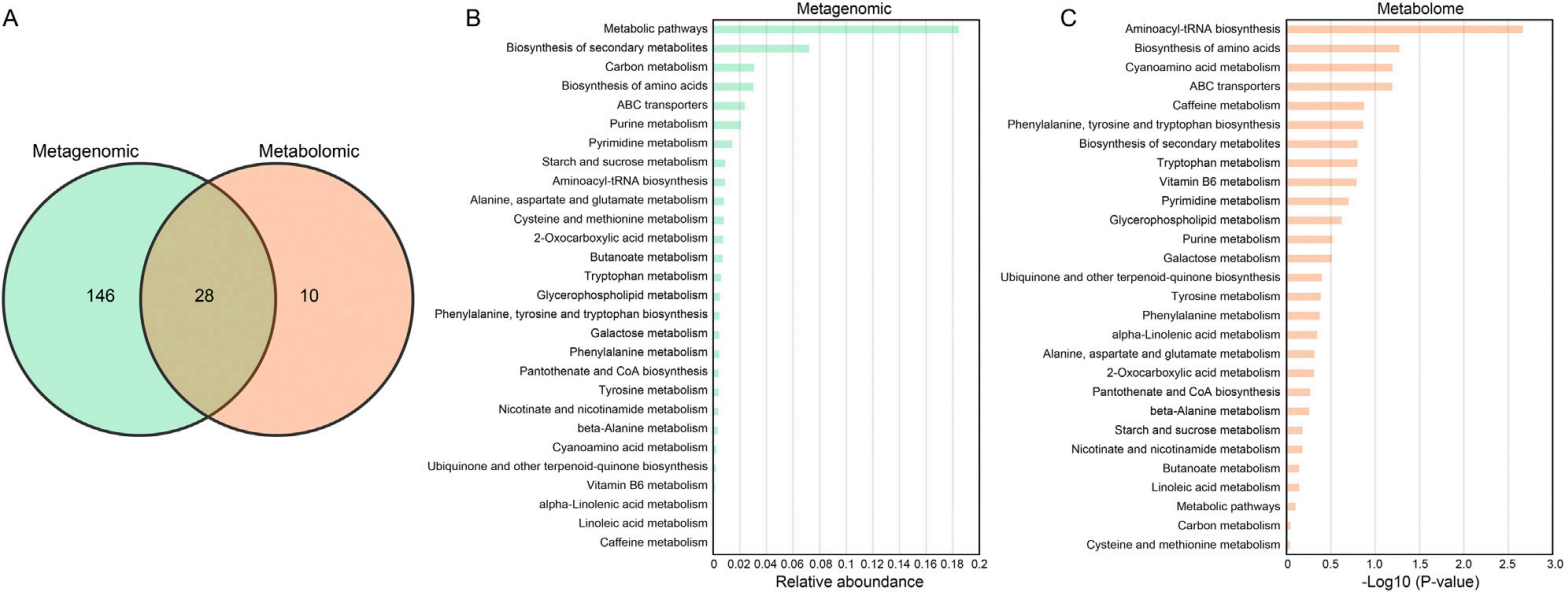
Integrated analysis of pathways enriched by microorganisms and differential metabolites. **(A)** Venn diagram illustrating the overlapping and specific metabolic pathway of microorganisms and metabolites. Relative abundance map of 28 overlapping KEGG pathways in **(B)** metagenome and **(C)** metabolome.

To explore the metabolic relationship between functional microbial communities and the production of fermentation products that are relevant to the taste and biochemical transformation of CTLs. The combined analysis of metagenomic and metabolomic was performed to analyze the changes in microbial community structure and metabolites during the fermentation process of CTLs. According to the KEGG enrichment analysis, a total of 174 and 38 metabolic pathways were identified in metagenome and metabolome, respectively. Notably, 28 metabolic pathways were co-enriched by both the metabolome and the macro group (Figure 5A). The relative abundance of these co-enriched metabolic pathways was shown in the bar chart (Figures 5B,C).

**FIGURE 6:**
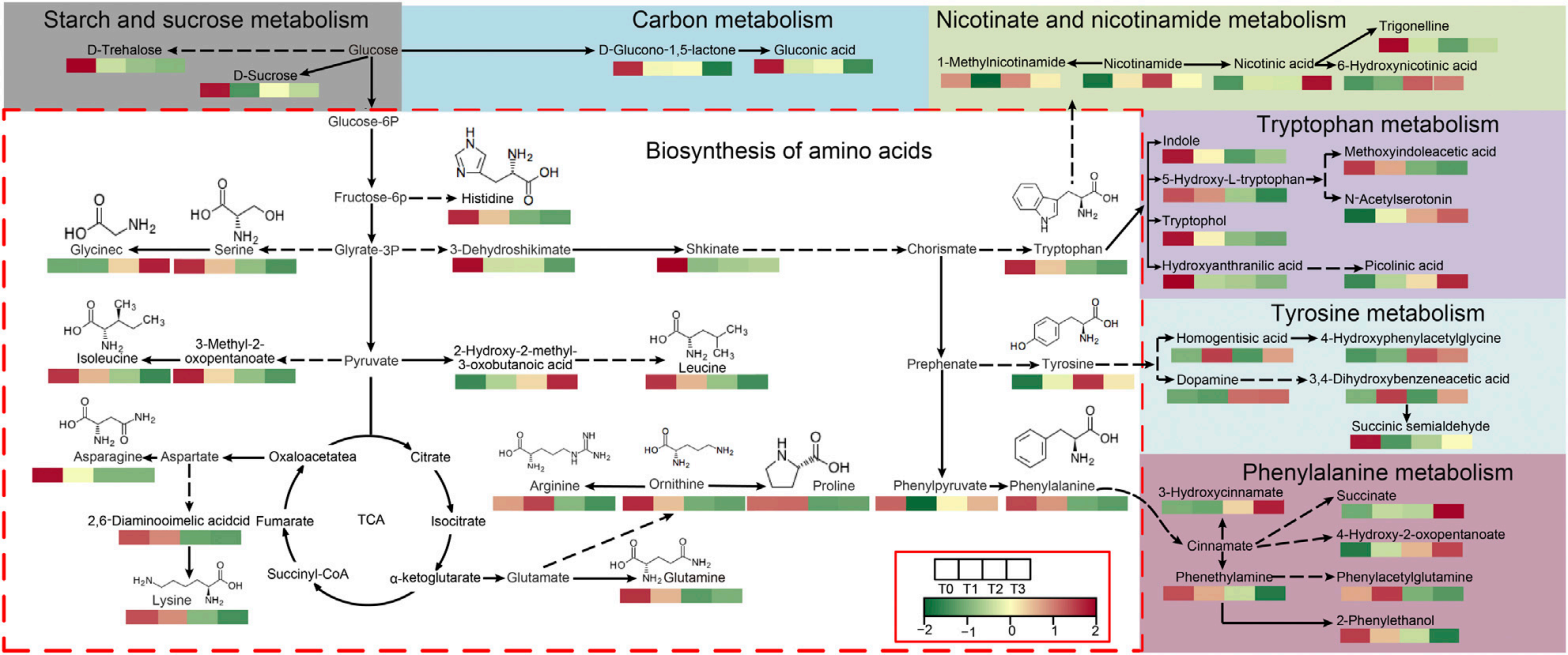
Map of 7 KEGG pathways associated with CTLs quality. Note: The proposed metabolic pathways were constructed based on experimental data analysis, with references to the KEGG PATHWAY Database and relevant literature.

Among these co-enriched pathways, a total of seven metabolic pathways are closely related to the formation of cigar quality, including amino acid biosynthesis, aromatic amino acid metabolic (tryptophan, tyrosine and phenylalanine), starch and sugar metabolic, carbon metabolic, and nicotinate and nicotinamide metabolism. Figure 6 illustrates the 7 metabolic pathways along with the 47 related metabolites involved in these pathways and their correlations. Notably, three overlapping metabolites across all comparison groups - L-Asparagine (amino acid biosynthesis), Shikimic acid (amino acid biosynthesis), and 3-Hydroxyanthranilic acid (tryptophan metabolism) - showed consistent downregulation (T0 *vs*. T1/T2/T3, Supplementary Table S3). Their decline aligns with the reduction of total nitrogen (4.76% to 3.62%) and nicotine (3.25% to 2.93%) in Figures 1D,E, suggesting microbial catabolism of amino acid precursors into volatile aroma compounds (e.g., phenylacetaldehyde).

**FIGURE 7:**
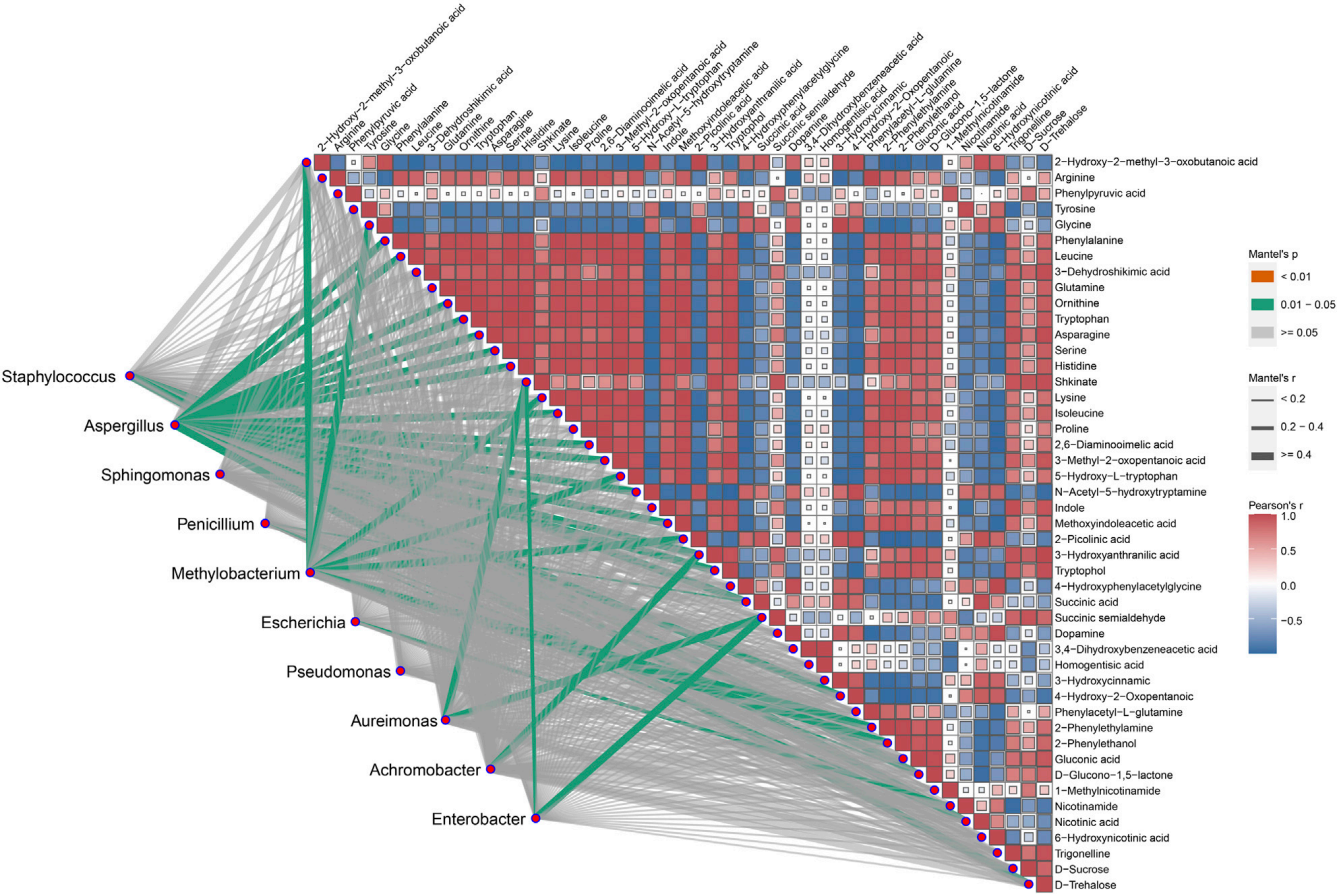
The correlation between the top 10 microbial community and the primary differential metabolites. The green line represents a significant correlation between the two omics, with the strength of the correlation increasing as the line thickness grows. The squares represent the different metabolites involved in the seven metabolic pathways, with the red squares depicting positive correlations, blue squares showing negative correlations, and uncolored boxes represent no relevant correlations.

Intra-group correlation analysis was performed within the 47 differential metabolites associated with the 7 metabolic pathways, and further inter-group correlation analysis was performed between these 47 differential metabolites and the top 10 microorganisms (Figure 7). In the intra-group correlation, significant correlations were found among the majority metabolites except four metabolites, namely, 3, 4-dihydroxybenzeneacetic acid, homogentisic acid, 1-Methylnicotinamide, and phenylpyruvic acid. Inter-group analysis showed that 8 dominant microbial genera, namely, *Staphylococcus*, *Aspergillus*, *Penicillium*, *Methylobacterium*, *Escherichia*, *Aureimonas*, *Achromobacter* and *Enterobacter*, were significantly associated with 29 metabolites. This result implies that these bacteria genera may serve as the functional bacteria involved in the metabolism of these metabolites.

## 4 Discussion

The color of CTLs is an important indicator for assessing cigar quality, as it reflects the internal quality of the cigars. In this study, CTLs exhibited a golden hue during the unfermented period (T0), but as fermentation time progressed, their color gradually deepened and eventually became a dark shade in the T2 and T3 periods (Figure 1A). This change in color is thought to be related to non-enzymatic reactions of monosaccharides such as Maillard reaction, as suggested by previous research (Liu et al., 2021). In our study, we observed variations in the levels of total soluble sugar, which can be broken down into monosaccharides. Notably, metabolites including D-Sucrose, D-Trehalose, Gluconic acid, and D-Glucono-1,5-lactone, which are produced through Starch and Sucrose metabolism and Carbon metabolism, were identified and their dynamics may contribute to these color-changing reactions.

The taste and aroma of CTLs are notable indicators for evaluating the quality of cigars, as they are associated with alkaloids, carbohydrates, and proteins (Arihara et al., 2016; Liu et al., 2021; Rivera and Tyx, 2021). This study examined the changes in starch, total soluble sugars, total nitrogen, and nicotine at four fermentation time points, which are closely related to the formation of alkaloids, carbohydrates, and proteins (Figures 1B–E). The results indicated that, compared to the control group at T0, there was a noteworthy increase in total soluble sugars at T1 during fermentation. However, a decrease in starch content and varying degrees of reduction in total nitrogen and nicotine were observed at T2 and T3. These findings are in line with Liu et al., 's 2015 study (2015), which suggested that microorganisms gradually degrade total nitrogen and nicotine into smaller molecules such as amino acids, organic acids, and volatile ammonia during the fermentation process, leading to a decrease in their content. Contrasting with Liu et al. (2015), who identified *Bacillus* as the primary starch degrader in Shiyan cigars, our study highlights *Staphylococcus* dominance—a divergence likely tied to Fujian’s distinct climate (higher humidity) (Philippot et al., 2024). Furthermore, most of the metabolites (14/22) produced from nicotinate and nicotinamide metabolism, tryptophan metabolism, tyrosine metabolism, and phenylalanine metabolism (Figure 6) showed an increasing trend in content during late-stage fermentation (T2, T3), further aligning with Liu et al. (2015) study conclusion.

The aroma components of CTLs mainly steam from the tobacco leaf and the fermentation process. Fermentation can contribute to the formation of tobacco aroma (Fan et al., 2023). In this study, a total of 18 metabolites were enriched in three aromatic amino acid metabolic pathways: phenylalanine, tryptophan, and tyrosine (Figure 6). Significant changes were observed in metabolites derived from these pathways during the fermentation process, such as phenylacetaldehyde, benzyl alcohol, and benzyl carbinol, which are known to play a role in cigar aroma formation. Additionally, metabolites, including 1-Methylnicotinamide, nicotinamide, nicotinic acid, and trigonelline, which are produced through nicotinamide metabolism, tryptophan metabolism, tyrosine metabolism, and phenylalanine metabolism, are thought to be linked to the sensory attributes of CTLs, particularly affecting the taste and flavor.

During the late stages of CTLs fermentation (T2 to T3), most quality-related indicators remained stable, suggesting that the fermentation process had largely plateaued. Visual characteristics such as color (Figure 1A) and key biochemical parameters—including starch, total soluble sugars, total nitrogen, and nicotine—showed minimal variation, indicating a state of equilibrium in chemical composition. Metabolomic analysis further supported this trend: the number of significantly different metabolites between time points decreased progressively, reflecting a diminishing influence of fermentation on metabolic profiles (Supplementary Figure S5). Notably, compounds associated with starch and sucrose metabolism, carbon metabolism, and amino acid biosynthesis—such as D-sucrose, D-trehalose, gluconic acid, D-glucono-1,5-lactone, proline, tyrosine, and phenylalanine—exhibited comparable levels at T2 and T3. However, extending fermentation to T3 may pose a risk of over-degradation of desirable flavor compounds. Although nicotine levels did not differ significantly between T2 and T3 (Figure 1E), a slight downward trend was observed. This subtle reduction could attenuate the characteristic pungency of tobacco, an essential sensory attribute that shapes the overall flavor profile and consumer acceptability. A parallel can be drawn with tea processing, where prolonged microbial fermentation leads to excessive degradation of polyphenols (Liu et al., 2018). These bioactive compounds are crucial for tea’s flavor, antioxidant capacity, and health benefits. Thus, just as in tea, over-fermentation in tobacco may compromise product quality and sensory attributes. These findings indicate that prolonging fermentation provides minimal additional benefits while potentially compromising critical quality attributes.

The activity of microorganisms during the fermentation process of CTLs is closely associated with changes in various metabolites, which directly influence cigar quality. Microbial communities in fermented CTLs vary depending on factors such as region, variety, and fermentation method. However, consistently dominant genera include *Staphylococcus*, *Aspergillus*, and *Pseudomonas* (Zheng et al., 2022b), finding that align with this study (Figure 3B). Specifically, *Staphylococcus* and *Aspergillus* exhibit significant positive correlations with carbohydrate metabolites D-Glucono-1,5-lactone and D-Trehalose, respectively (Figure 7). This indicates that *Aspergillus* and *Staphylococcus* drive carbon metabolic and starch and sugar metabolic through enzymes like α-amylase and glucoamylase, facilitating starch degradation and promoting the formation D-Glucono-1,5-lactone and D-Trehalose (Lakshmi et al., 2013; Yun et al., 2020). Moreover, *Aspergillus* shows a strong correlation with amino acids such as phenylalanine, leucine and ornithine, suggesting its involvement in amino acid metabolic pathways that contribute to aroma development in CTLs.

While this study has made initial strides in identifying key microbial taxa and their correlations with metabolite dynamics through multi-omics approaches, it further generates a novel testable hypothesis: that the dynamic co-occurrence of *Staphylococcus*, *Ascomycota* and *Penicillium* between T1 and T2 stages establishes a synergistic metabolic network crucial for the degradation of macromolecules (e.g., starch, nicotine) and the concurrent synthesis of key aroma compounds. However, the functional roles of these microorganisms and the underlying enzymatic mechanisms have not been experimentally validated. Future research should focus on constructing synthetic microbial consortia based on these dominant genera to verify their contributions to flavor and quality development. Additionally, targeted inoculation experiments and mechanistic studies—particularly those measuring specific enzyme activities (e.g., amylases, proteases, and nicotine-degrading enzymes) and quantifying the resulting metabolic products—are essential to elucidate the causal relationships and biochemical pathways involved. Such efforts would not only validate the proposed hypothesis but could also significantly enhance the fermentation process of cigar tobacco leaves and ultimately improve their overall quality.

## 5 Conclusion

This study demonstrate that the T2 stage achieves an optimal balance between microbial equilibrium and metabolite stabilization, providing a scientific foundation for refining industrial fermentation processes. More importantly, it proposes a novel mechanistic hypothesis: the microbial equilibrium observed at the T2 stage signifies not just taxonomic stability but also functional convergence, driving the optimal transformation of metabolites essential for cigar quality. This hypothesis provides a foundation for developing synthetic microbial communities specifically tailored to improve product quality.

## Data Availability

The datasets presented in this study can be found in online repositories. The raw reads were submitted to NCBI SRA (Sequence Read Archive, http://www.ncbi.nlm.nih.gov/sra/) under the accession number PRJNA1190085, while the generated datasets is provided within the manuscript or supplementary information files.

## Supplementary material

SUPPLEMENTARY FIGURE S1 The beta diversity of the CTLs microbiota is based on the binary jaccarad distance and bray curtis distance.

SUPPLEMENTARY FIGURE S2 The alpha diversity of the CTLs microbiota is based on the Chao-2 and pielou_evenness index, respectively. * *P* < 0.05, ** *P* < 0.01.

SUPPLEMENTARY FIGURE S3 The HCA of the 1801 metabolites of CTLs samples.

SUPPLEMENTARY FIGURE S4 The score charts of the OPLS-DA model for three comparisons.

SUPPLEMENTARY FIGURE S5 Bar chart showing the number of differentially expressed metabolites (DEMs) in three comparison groups.
