# Supplementary figures and images for: Integrated metagenomics and metabolomics analysis reveals dynamic changes of microbiota and metabolic profile during fermentation of cigar tobacco (Nicotiana tabacum L.) leaves
Source: Front Genet. 2025 Nov 10;16:1662815. doi: 10.3389/fgene.2025.1662815 (PMC12640762; doi:10.3389/fgene.2025.1662815)

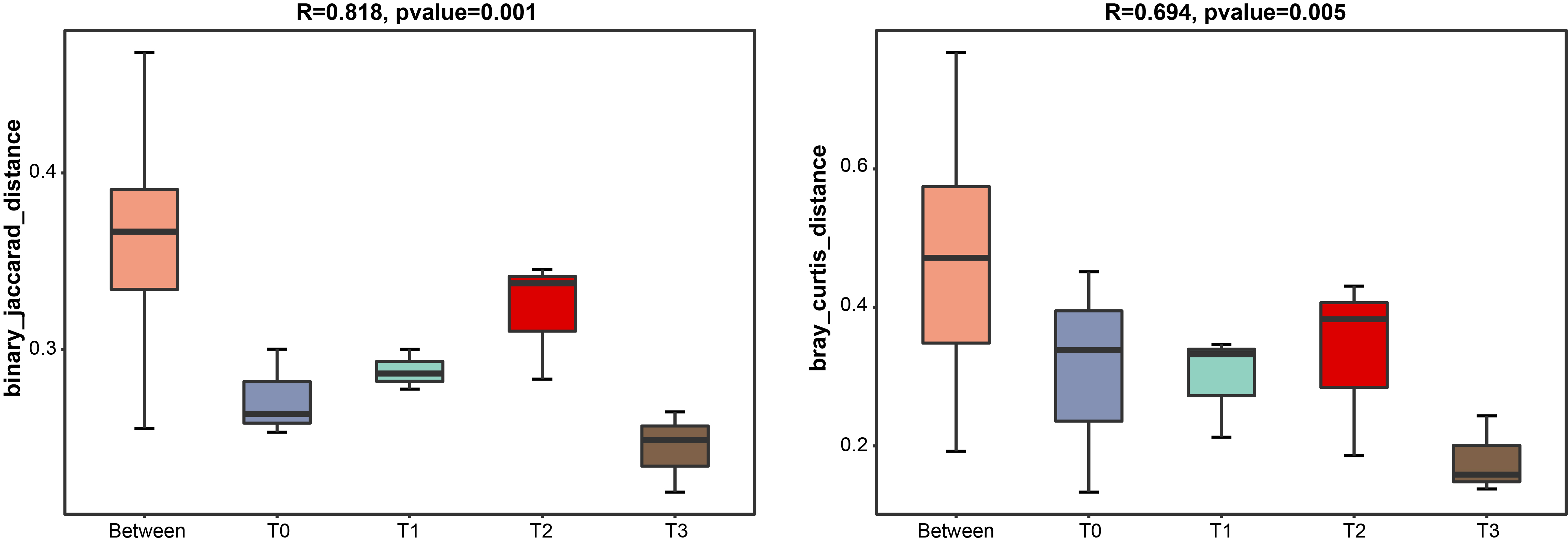

Supplement: Supplementary file 4 [file Image1.jpeg]

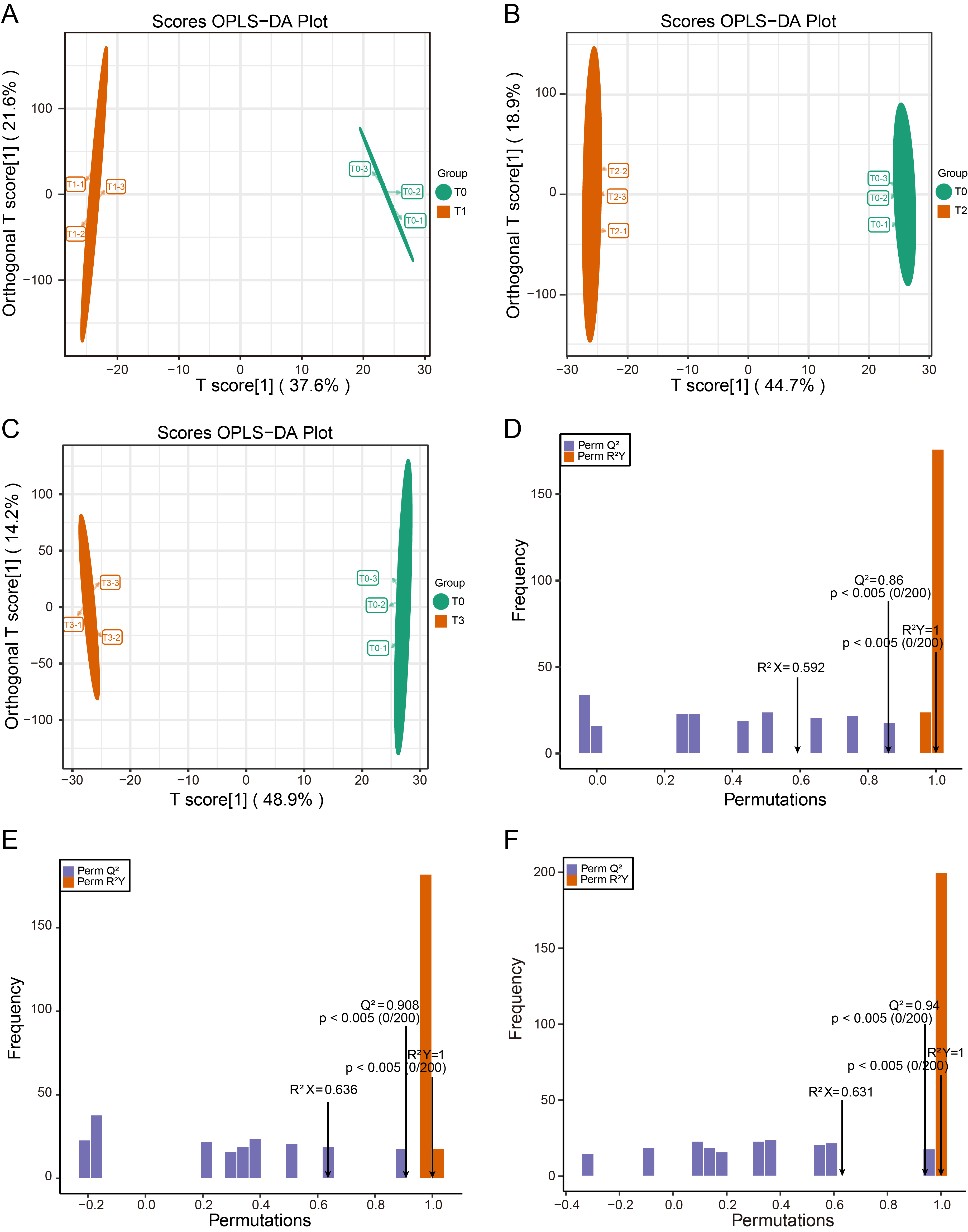

Supplement: Supplementary file 5 [file Image4.jpeg]

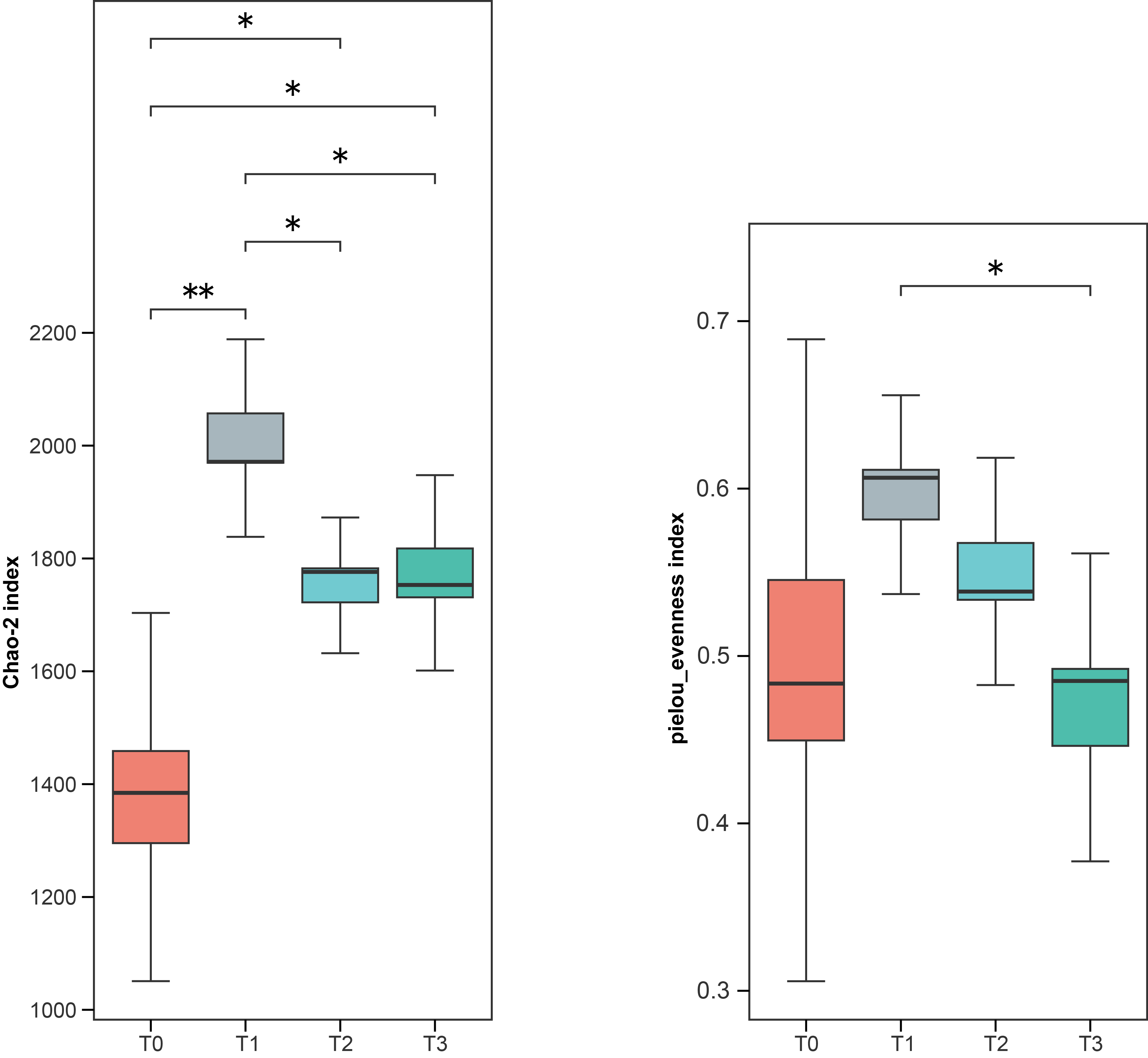

Supplement: Supplementary file 6 [file Image2.jpeg]

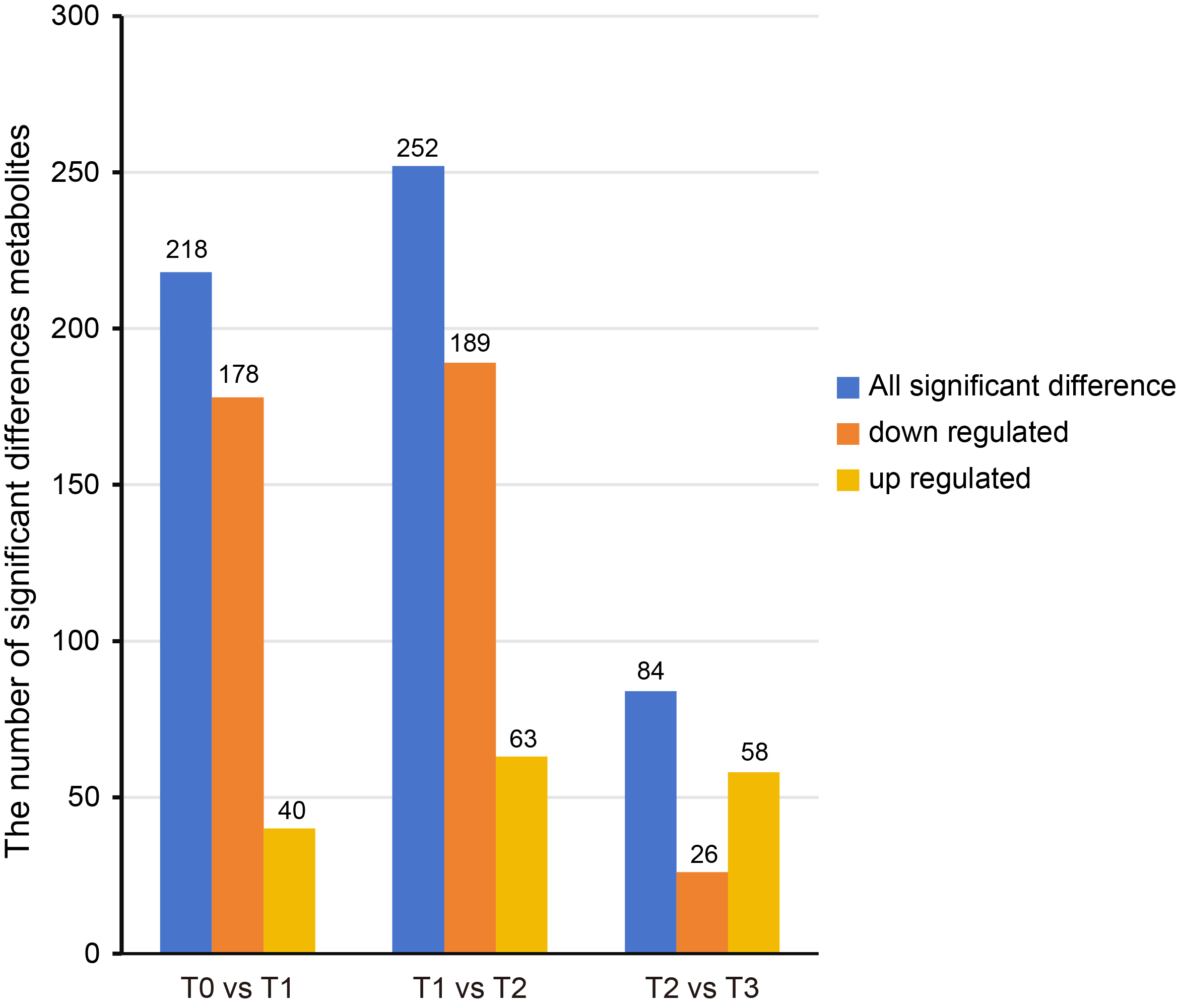

Supplement: Supplementary file 7 [file Image5.jpeg]
